# Neonatal alloimmune thrombocytopenia caused by anti-HPA antibodies in pregnant Chinese women: a study protocol for a multicentre, prospective cohort trial

**DOI:** 10.1186/s12884-017-1453-y

**Published:** 2017-08-31

**Authors:** Li Chen, Zhiwei Liu, Tiemei Liu, Xianjun Ma, Meiying Rao, Yongjun Wang, Bo Sun, Wen Yin, Jun Zhang, Beizhan Yan, Xiaojuan Li, Qiushi Wang, Lei Zhang, Jun Wen, Fenghua Liu, Peng Wang, Yaming Wei, Yuanshuai Huang, Jiang Wu, Yi Guo, Yinlan Kang, Xiaochuan Song, Xiangfu Liu, Genling Zhang, Tingting Xie, Yonggeng Chen, Xiaojing Zeng, Zhongjun Li

**Affiliations:** 1 Department of Blood Transfusion, The Second Affiliated Hospital, The Third Military Medical University, Chongqing, People’s Republic of China; 2 Department of Blood Transfusion, Sir Run Run Shaw Hospital, School of Medicine, Zhejiang University, Hangzhou, 310016 People’s Republic of China; 3 Department of Blood Transfusion, China-Japan Union Hospital of Jilin University, Changchun, China; 4 Department of Blood Transfusion, Qilu Hospital of Shangdong University, Jinan, China; 5 Derpartment of Blood Transfusion, The Second Hospital of Nanchang University, Nanchang, China; 6 Department of Blood Transfusion, The Second Xiang Ya Hospital of Central South University, Changsha, China; 7 Department of Blood Transfusion, The Affiliated Hospital of Qingdao University, Qingdao, China; 8 Department of Blood Transfusion, Xijing Hospital of Fourth Military Medical University, Xi’an, China; 9 Department of Blood Transfusion, The First Affiliated Hospital of Bengbu Medical College, Bengbu, China; 10 Department of Blood Transfusion, Henan Provincial People’s Hospital, Zhengzhou, China; 11 Department of Blood Transfusion, The Second Hospital of Lanzhou University, Lanzhou, China; 12 Department of Blood Transfusion, Shengjing Hospital of China Medical University, Shenyang, China; 13 Department of Blood Transfusion, Affiliated Beijing Chaoyang Hospital, Capital Medical University, Beijing, China; 14 Department of Blood Transfusion, The People’s Hospital of Xinjiang Autonomous Region, Wulumuqi, China; 15 Department of Blood Transfusion, The First Affiliated Hospital of Harbin Medical University, Harbin, China; 16 Department of Blood Transfusion, Peking University First Hospital, Beijing, China; 17 Department of Blood Transfusion, Guangzhou First People’s Hospital, Guangzhou, China; 18 Department of Blood Transfusion, The Affiliated Hospital of Southwest Medical University, Luzhou, China; 19 Department of Blood Transfusion, Renji Hospital, Shanghai Jiaotong University School of Medicine, Shanghai, China; 20 Department of Blood Transfusion, The First Affiliated Hospital/School of Clinical Medicine of Guangdong Pharmaceutical University, Guangzhou, China; 21 Department of Blood Transfusion, MCH Hospital of Yinchuan City, Yinchuan, China; 22 Department of Blood Transfusion, The First Hospital of Xinjiang Medical University, Wulumuqi, China; 23 Department of Blood Transfusion, The Third Affiliated Hospital, SUNYAT-SEN University, Guangzhou, China; 24 Department of Blood Transfusion, The Affiliated Hospital of Guizhou University, Guiyang, China

**Keywords:** Human platelet antigen, Neonatal Alloimmune thrombocytopenia, Human leukocyte antigen

## Abstract

**Background:**

Neonatal alloimmune thrombocytopenia (NAIT), caused by maternal antibodies raised against alloantigens carried on foetal platelets, is a very common haematological abnormality in newborns worldwide. However, baseline data on NAIT in China are lacking. Therefore, this study seeks to explore the incidence of alloantibody against the human platelet antigen (HPA) in pregnant women and its associations with NAIT in China.

**Methods:**

A multicentre, prospective cohort study design will be used, and 55,497 pregnant women will be recruited for the first screening of the anti-HPA antibody at 12 to 28 weeks of gestational age. Subjects who are positive in the first screening for the anti-HPA antibody will be included in the exposure group. Re-tests of the antibody titre, antigen-specificity and genotyping of HPA and HLA will be conducted during admission. A ratio of 1:1 paired individuals with the same ethnicity and parity but testing negative for the anti-HPA antibody will be randomly selected to be included in the non-exposure group. NAIT will be diagnosed in the newborns on day one of the birth. The HPA of the neonates in the exposure group will also be genotyped by sequencing. Associations of maternal HLA with the occurrence of the anti-HPA antibody and correlation of the severity of NAIT with the titre of the anti-HPA antibody will be further analysed.

**Discussion:**

The study is expected to provide baseline data on NAIT in China. Besides, we hope to find out a population who expresses particular HLA molecules has significant higher risk of HPA alloimmunization in Chinese individuals. We also hope to find a Chinese-specific cut-off antibody titre for the prediction of the severity of NAIT and to provide a means to evaluate the necessity of antenatal treatment.

**Trial registration:**

ClinicalTrials.gov: NCT02934906 (date registered: 13.10.2016).

## Background

Neonatal alloimmune thrombocytopenia (NAIT) is caused by maternal immunoglobulin G (IgG) antibodies raised against incompatible human platelet alloantigen (HPA) carried on foetal platelets. Foetal platelets are sensitized by anti-HPA IgG and destroyed by the monocyte-phagocyte system [1]. Although many cases are mild, NAIT is one of the most frequent causes of severe thrombocytopenia (platelet count <50 × 10^9^/L) and intracranial haemorrhage (ICH) [2].

The HPA is highly polymorphic. Currently, 35 HPA antigens have been indexed in the Immuno Polymorphism Database (IPD) (http://www.ebi.ac.uk/ipd/hpa/). The antigen type of HPA varies according to ethnicity, which directly causes differences in the incidence and antigen-specificity of anti-HPA antibodies and the morbidity of NAIT. In Caucasian populations, the majority (>75%) of NAIT cases are due to fetomaternal incompatibility of HPA-1a [3]. In contrast, anti-HPA4b and anti-HPA5b are the main causes of NAIT in the Japanese population [4]. The incidence of NAIT is 1 in 1000 in Caucasians [5] and 1.5 in 1000 in Japanese subjects [4].The consequence of NAIT in women with HPA-4b or HPA-5b is less severe than that in Caucasian women with anti-HPA-1a. Generalized purpura and even ICH have been found in HPA-1a-incompatible neonates [2]. In the Japanese population, purpura has been found to develop rarely in HPA-4b-incompatible infants and not to develop in HPA-5b-incompatible infants [4].

It has also been reported that the presence of HPA antibodies is tightly associated with human leukocyte antigen (HLA). Among these antibodies, the most well known is HLA-DRB3*0301, which is strongly associated with alloimmunization to HPA-1a [6, 7]. The immunization rates also correlate linearly with the number of pregnancies. The anti-HPA antibody (mainly against HPA-4b and HPA-5b) has been found in only 0.19% (95% CI: 0.11-0.28%) of women in their first pregnancy and in 1.97% (95%CI: 1.41-2.54%) of women in their fourth or subsequent pregnancies [4].

Few data have been reported regarding the incidence and antigen-specificity of the anti-HPA antibody, the morbidity of NAIT and clinical outcomes among Chinese patients. Therefore, this study seeks to provide these baseline data on NAIT caused by anti-HPA antibodies in pregnant Chinese women.

## Methods/design

This study is a nationwide, prospective, non-interventional, multicentre cohort study. The objectives are listed in Table 1. The survey and data collection will provide baseline data on NAIT in China and help form an appropriate clinical screening procedure.

**Table 1:** Study objectives

Primary objectives	
• The positive incidence of the anti-HPA antibody in pregnant Chinese women.	
• The morbidity of NAIT in anti-HPA antibody-positive pregnant women in China.	
Secondary objectives	
• Antigenic specificity of anti-HPA antibodies.	
• Association of the anti-HPA antibody with HLA genotype.	
• The relationship between the titre of the anti-HPA antibody and the clinical symptom severity of NAIT.	

This study is non-interventional. The assignment of subjects to antenatal treatments (such as intravenous immunoglobulin and intrauterine fetal platelet transfusion) or a particular delivery planning is not decided in advance but instead falls within current practice.

Study recruitment started primarily at the sponsor centre at The Second Affiliated Hospital of The Third Military Medical University on 1 November 2016. All other centres initiated consecutive recruitment before the end of January 2017. Recruitment is expected to be completed before December 2018.The goal is to enrol 55,497 women at 12-28 weeks of gestation.

The study subjects will be pregnant Chinese women and their newborns. Inclusion and exclusion criteria are listed in Table 2. Subjects with NAIT in a previous pregnancy or a positive family history of first degree relatives will not be excluded but will be analyzed hierarchically.

**Table 2:** Inclusion and exclusion criteria

Inclusion criteria
• Pregnant women 18–50 years of age
• Pregnant women at 12-28 weeks of gestation between 1 January 2017 and 31 December 2018.
• Willingness of the mother to give birth in a participating hospital
• Willingness to participate in the study
Exclusion criteria
• Pregnant women unwilling to participate in the study
• Pregnant women planning to prematurely terminate the pregnancy

### Determination of sample size

For one of the primary objectives, the investigation of the positive incidence of the anti-HPA antibody, we calculated the sample size by using a formula for cross-sectional studies: $n={Z}_{\alpha /2}^2p\left(1-p\right)/{\delta}^2$. On the basis of an estimated positive rate of the anti-HPA antibody of 0.19% according to a study in Japanese patients [4], an α error of 0.05 and an β error of 0.2, the estimated sample size should be 55,497 to offset a dropout rate of 10%. For the other primary objective, the investigation of the morbidity of NAIT in anti-HPA antibody-positive pregnant women, we calculated the sample size by using a formula for cohort studies: $n={\left({Z}_{\alpha}\sqrt{2\overline{p}\overline{q}}+{Z}_{\beta}\sqrt{p_1{q}_1+{p}_0{q}_0}\right)}^2/{\left({p}_1-{p}_0\right)}^2$. On the basis of an estimated morbidity of NAIT of 16.7% in the anti-HPA antibody-positive group and 2.5% in the antibody non-exposure group according to the Japanese data [4], an α error of 0.05 and an β error of 0.1, the estimated sample size of anti-HPA antibody-positive subjects should be 98 if a 10% dropout rate is considered. Because the estimated positive incidence of the anti-HPA antibody is 0.19%, the estimated sample size should be 51,578. To ensure that both of these two primary objectives can be addressed in this study, 55,497 pregnant women will be recruited. A sample size review will be performed after the first 10,000 recruitments.

### Recruitment and informed consent

The study is being conducted at 24 Grade-A Tertiary Hospitals in China. Subjects testing positive in the anti-HPA antibody test at 12-28 weeks of gestation will be included in the exposure group. A ratio of 1:1 paired individuals with the same ethnicity and parity but negative in the anti-HPA antibody test will be randomly selected to be included in the non-exposure group. The investigators will inform the participants about all aspects about the trial. The informed consent will include permission to collect blood samples from the mothers and their infants for antibody screening and genotyping of HPA and HLA, gathering data from their medical records and the storage of biological samples for a maximum of 10 years for additional analyses related to the current study. The participants will be informed that trial participation is voluntary and that they are free to withdraw at any time. All the investigators are aware of the guidelines for good clinical practice [8].

### Study procedure and data collection

All healthy pregnant women receiving regular care at the obstetrical outpatient department of participating hospital will be counselled and asked at 12-28 weeks of gestation to participate in this study. General information will be collected and the anti-HPA antibody evaluation will be conducted after informed consent is received. The test of the antibody titre and antigen-specificity will be conducted at the production admission if the anti-HPA antibody test is positive. Genotyping of HPA will be further completed for anti-HPA antibody-positive mothers and their infants. Genotyping of HLA will be performed for mothers in both the exposure and non-exposure groups. Platelets of their newborns at day one of the birth will be counted, NAIT and ICH will be diagnosed.

### General information

General data, maternal health history and delivery records will be obtained from the medical records. Confounding factors affecting the platelets of the newborns will be collected by telephone interview. All of the information will be recorded in the Case Report Form (CRF).

### Platelet counting

Ethylenediaminetetraacetic acid (EDTA) anti-coagulated venous blood and cord blood will be used for the platelet counting in the mothers and their babies, respectively.

### Anti-HPA antibody screening, identification and titration

The Monoclonal Antibody Solid Phase Platelet Antibody Test (MASPAT) Kit will be used in the screening of potential anti-HPA antibody-positive pregnant women [9]. The anti-HLA antibody and the anti-HPA antibody will be further distinguished by chloroquine-treated (i.e., HLA-depleted) platelets [4]. Two-fold serum dilutions in phosphate-buffered saline from undiluted to one in 1024 will be done to determine the titre of the anti-HPA antibody. Antigen specificity will be confirmed by LIFECODES Pak Lx (Immucor) [10].

### Diagnosis and grading of NAIT

NAIT is defined as a platelet count in the neonates of less than 150 × 10^9^/L and the HPA is incompatible with the mother. When the platelet count is less than 50 × 10^9^/L, we will grade the NAIT as severe. These cases are distinguished from cases of moderate (platelet count 50 × 10^9^ - 100 × 10^9^/L) or mild NAIT (platelet count 100 × 10^9^-150 × 10^9^/L) [5].

### Diagnosis of ICH

For all newborns with thrombocytopenia, an ultrasound diagnosis will be further performed to determine ICH.

### HPA and HLA genotyping

HPA genotyping will be performed with BioArray HPA BeadChips (IMMUCOR) [11]. HLA genotyping of DRB1, DRB3, DRB4, DRB5, DPB1 and DQB1 will be conducted through PCR-SBT (polymerase chain reaction - sequence based typing) (SBT*excell*erator®).

### Follow-up

There will be two follow-up visits after recruitment. One will be at the end of pregnancy before delivery, in which re-tests of the antibody titre, antigen-specificity and genotyping of HPA and HLA will be conducted in the mothers. The second follow-up will be after delivery, in which platelet counting and HPA genotyping of the newborns will be performed and NAIT will be diagnosed and graded.

### Variable and potential confounders

The primary outcome variables will be the titre of the anti-HPA antibody of the mothers and the platelet count of the newborns. The secondary outcome variables will include the pregnancy number. The potential confounding variables that will be measured will include nationality, maternal history (e.g., parity, still birth and abortion), allogenic platelet transfusion history and diseases leading to a decreased platelet count in the mother (e.g., idiopathic thrombocytopenic purpura, eclampsia and gestational diabetes mellitus) and in the newborns (e.g., intrauterine growth retardation, intrauterine infection and neonatal asphyxia).

### Statistical analysis

The data will be analysed using SPSS V18 (SPSS, Chicago, Illinois, USA). 95% confidence levels will be set to test for significance. Descriptive statistics will be used to analyse the general data, the positive incidence of the anti-HPA antibody and the morbidity of NAIT. The frequency of thrombocytopenia between groups will be compared by using the χ^2^ test. The χ^2^ test will also be used in the analysis of the associations between the pregnancy number (or mother HLA genotype) and the incidence of the anti-HPA antibody. The correlations between the titre of the anti-HPA antibody and the severity of NAIT will be analysed by using the multiple regression analysis.

## Discussion

To the best of our knowledge, this will be the first large Chinese cohort study to prospectively investigate the incidence of the anti-HPA antibody in pregnant Chinese women and the morbidity of NAIT in their newborns. Currently, HPA antibodies are not routinely screened for in every pregnant woman, and NAIT is always diagnosed after the birth of the first affected child. Therefore, antenatal treatment can be offered only in the subsequent pregnancies to avoid the recurrence of severe FNAIT [12]. The findings of this study should not only provide baseline data on NAIT in Chinese newborns but also aid in evaluating the necessity of the screening of the anti-HPA antibody in pregnant Chinese women.

A discrepancy between the expected and the actual rates of HPA alloimmunization in pregnancy has previously been observed. For example, in Caucasian women, 2% have the HPA-1b/b genotype, and 98% are married to a man with the HPA-1a gene. However, only 10% of HPA-1a-incompatible pregnancies result in alloimmunization of HPA-1a [13, 14]. One explanation for this discrepancy is that HPA-1a alloimmunization is strongly associated with the HLA-DRB3*01:01 [7, 13, 15]. The HPA-1a alloepitope presented by HLA-DRB3*01:01 has been further identified by using CD4 T cell clones from alloimmunized HPA-1b/b mothers [16]. Except for HLA-DRB3*01:01, HLA-DQ*02:01 has also been reported to be associated with HPA-1a alloimmunization [6], and HLA-DRB1*15:01 has even been found to be inversely linked to FNAIT occurrence [17].The chance of incompatibility with other HPA antigens is greater than that with HPA-1a. However, even fewer cases of FNAIT have been reported to be caused by antibodies against these antigens [1]. Given the large differences in the HLA gene background, little is known about the association of HLA alleles and HPA alloimmunization in Chinese individuals. Our study will also evaluate this key point.

Although HLA-DRB3*01:01 is highly associated with the incidence of HPA-1a alloimmunization, it cannot be used to differentiate high- and low-risk pregnancies or to guide antenatal treatment in affected families [18]. Instead, the titre of the anti-HPA antibody has been reported to be a much better indicator of the severity of NAIT. The levels of the anti-HPA antibody have significant correlations with the risk [19] and the severity of neonatal thrombocytopenia [20]. When the antibody titre is ≥1:64 for HPA-5b in Japanese [19] and ≥1:32 [20] for HPA-1a in Caucasians, NAIT is significantly more severe. In this study, we also hope to find a Chinese-specific cut-off antibody titre for the prediction of the severity of NAIT and to provide a means to evaluate the necessity of antenatal treatment.

## Abbreviations

CRF: Case Report Form
EDTA: Ethylenediaminetetraacetic acid
HLA: human leukocyte antigen
HPA: human platelet antigen
ICH: intracranial haemorrhage
IPD: Immuno Polymorphism Database
MASPAT: Monoclonal Antibody Solid Phase Platelet Antibody Test
NAIT: Neonatal alloimmune thrombocytopenia
